# Utilization of Oxford Nanopore Technology for human infectious disease detection and surveillance in Africa: a scoping review

**DOI:** 10.1099/acmi.0.001020.v3

**Published:** 2025-07-15

**Authors:** Kristen Bastug, Uzal Umar, Brooke Olson, Tina Slusher, Christopher Faulk, Mark Okolo, Stephen Oguche, Beth K. Thielen

**Affiliations:** 1 Department of Pediatrics, Division of Pediatric Infectious Diseases, University of Minnesota, Minneapolis, MN, USA; 2 Department of Medical Microbiology and Parasitology, Faculty of Clinical Sciences, College of Health Sciences, University of Jos, Jos, Plateau State, Nigeria; 3 Health Sciences Libraries, University of Minnesota, Minneapolis, MN, USA; 4 Department of Pediatrics and Division of Critical Care, University of Minnesota, Minneapolis, MN, USA; 5 Department of Pediatrics and Division of Critical Care, Hennepin County Medical Center, Minneapolis, MN, USA; 6 Department of Animal Science, College of Food, Agricultural and Natural Resources Science, University of Minnesota, Saint Paul, MN, USA; 7 Department of Medical Microbiology, University of Jos and Jos University Teaching Hospital, Faculty of Basic Clinical Sciences, College of Health Sciences, Jos, Plateau State, Nigeria; 8 Department of Paediatrics, Faculty of Clinical Sciences, College of Health Sciences, University of Jos and Jos University Teaching Hospital, Jos, Plateau State, Nigeria

**Keywords:** clinical laboratory techniques, molecular epidemiology, molecular diagnostic techniques, nanopore sequencing, rapid diagnostic tests

## Abstract

**Background.** Nanopore‐based sequencing by Oxford Nanopore Technologies (ONT) offers rapid, cost‐effective and portable sequencing. As an emerging technology, ONT must be evaluated for efficacy and practical application in both high‐ and low‐resource settings. This scoping review (SR) aimed to (1) describe how nanopore technology is used in Africa for surveillance and diagnosis of human infectious diseases, (2) describe how nanopore technology aids in the real-time detection of infectious pathogens in Africa and (3) identify challenges and opportunities for utilizing nanopore technology in Africa to study infectious diseases.

**Methods.** This SR followed the Joanna Briggs Institute Reviewer’s Manual framework for SRs. English language studies published from 1 January 2008 to 30 April 2024 that used ONT on human specimens collected in Africa and targeted ≥1 microbial agent were included. Searches were performed in Embase, Medline, PubMed, CINAHL and the Cochrane Library. The protocol was publicly available on the Open Science Framework Bastug *et al.* (Nanopore Sequencing for Infectious Diseases Surveillance and Diagnostics in Africa: a Scoping Review 2024) prior to data collection. Two independent reviewers screened studies using Covidence, and data was extracted using a custom REDCap instrument. Descriptive statistics and data visualization were performed in Microsoft Excel.

**Results.** One thousand one hundred sixty-two studies were identified and 93 (8%) underwent full-text review. The portable MinION Mk1B was the most common ONT device (65% of studies). Eighty-eight studies analysed specimens from a single African country. Of these, 45% were sequenced in the same country, 7% in a different African country and 11% in a non-African country, while 32% did not specify the location. Specimen types included direct patient specimens (62%) and cultured isolates (35%), or a combination of both. Blood, serum or plasma was most common (35%), followed by naso- or oropharyngeal specimens (27%). Forty-four studies used ONT during an active infectious disease outbreak, 25 of which studied severe acute respiratory syndrome coronavirus 2 (SARS-CoV-2). Seventy-two studies used ONT for genomic surveillance of infectious pathogens or antibiotic resistance genes, and one study used ONT for a direct clinical application. African-affiliated authors were included as first, middle and last authors in 46% of studies, and 15% were published by entirely African-affiliated teams. Ten studies published information on workflow timeline, and five studies published the per-specimen cost.

**Conclusions.** ONT can enable timely and affordable sequencing in African countries as demonstrated through a small number of studies that accomplished these goals individually. Most studies used ONT for genomic surveillance of pathogens or antimicrobial resistance genes, while only one study used ONT directly for a real-time clinical application. A small number of studies described a short interval between specimen collection and sequence result, supporting that clinical applications are possible. There is a need for improved reporting of ONT methodology including pipeline timelines, cost, use of barcoding, flow cell models and the use of negative controls. Publications that provide these details will enhance reproducibility and support the development of new studies using ONT for the diagnosis and surveillance of infectious diseases in low-resource settings.

**Impact Statement**

Molecular diagnostic approaches for the detection of infectious pathogens have advanced and now include long-read nanopore sequencing developed by Oxford Nanopore Technologies (ONT). This technology offers portability, the potential for reduced cost and faster turnaround time from sample collection to results compared with existing methodologies. These factors may facilitate the expansion of molecular sequencing into low-resource settings, but the feasible and practical use of ONT in Africa has not been described. This scoping review describes the current use of ONT sequencing in Africa for surveillance and diagnosis of human infectious diseases in regard to common pathogens, study populations and laboratory approaches. Researchers seeking to use ONT in resource-limited settings for infectious diseases research will gain insights into current applications and methodologic approaches for using this technology. The research gaps identified in this review form a strong framework to guide future studies using ONT in resource-limited settings.

## Data Summary

The authors confirm that all supporting data, code and protocols have been provided within the article or through supplementary data files.

## Introduction

**Fig. 1.**
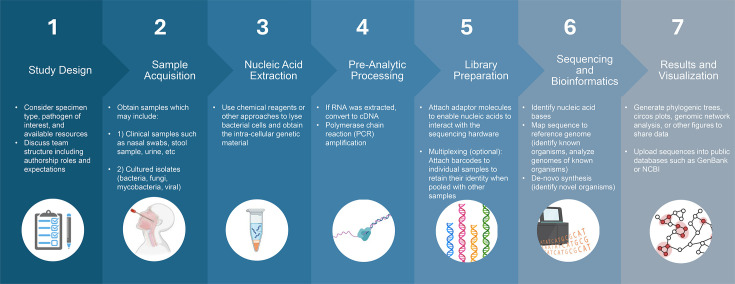
Typical steps in a NGS workflow. The approach begins with study design (left side) and ends with generation of results (right side).

Genomic sequencing technology has significantly advanced since the development of Sanger sequencing in the 1970s. Whereas Sanger sequencing analysed DNA fragments generated from a single DNA template, next-generation sequencing (NGS) approaches simultaneously sequence millions of nucleic acid molecules. Nucleotide (NT) sequences from many organisms within the same sample can be analysed through a process termed ‘metagenomic’ NGS. The general NGS workflow (Fig. 1) begins with a consideration of the study design, followed by sample acquisition (e.g. a direct patient specimen or cultured isolate). RNA or DNA is extracted from the sample via different approaches, which may include the use of chemical reagents to lyse open cells and obtain intra-cellular material. Pre-analytic processing is then performed, which may include the conversion of RNA into cDNA and/or polymerase chain reaction (PCR) amplification to increase the abundance of the nucleic acid target of interest. The cDNA then undergoes ‘library preparation’ which can involve attaching adapter molecules to facilitate interaction with the sequencing platform and/or attaching unique sequences termed ‘barcodes’ to track individual samples within a larger pool of specimens. The sample or pooled specimen is then placed on the sequencer to determine the sequence of nt bases. Bioinformatic tools are used either simultaneously during sequencing or in post-processing analysis. This may include (1) mapping the obtained sequences to existing reference genomes of known organisms to detect the presence of an organism or investigate its whole-genome sequence or (2) using the sequence data to construct a new genome from scratch in a process referred to as ‘*de novo* assembly’. The genome sequences can be uploaded into public databases such as GenBank or the European Nucleotide Archive, and results can be visualized in the form of phylogenetic trees, circos plots, genome network analyses and more.

The options for NGS sequencing have expanded due to the development of newer technologies that can sequence long, continuous stretches of nucleic acids. Two prominent long-read sequencing approaches include nanopore sequencing [developed by Oxford Nanopore Technology (ONT)] and single-molecule real-time technology (developed by PacBio). ONT offers potential advantages for use in low- and middle-income countries (LMICs), particularly the availability of ONT as a small, portable device (the MinION sequencer), lower cost and real-time data generation. The capital equipment cost of the MinION is less than $5,000 USD, compared with over $100,000 for the equipment cost of other sequencing platforms. This may enable ONT to be used in laboratories with smaller budgets. Additionally, NGS platforms often achieve cost reductions by analysing samples in large batches of samples, but this approach is ill-suited for meeting the needs of clinicians who often need to identify pathogens in singular patients within 24–48 h. During active sequencing, the MinION can align sequence reads to known pathogens and report results while sequencing is occurring, prior to the completion of the entire sequencing run. This method of real-time reporting is unique to ONT and can be incorporated into custom laboratory workflows to answer clinical questions.

### Nanopore sequencing offers a unique approach for NGS

The underlying mechanism of nanopore sequencing and its associated workflow offers customization options that enable flexibility for different settings and experimental goals. ONT sequencing functions by passing individual nucleic acid molecules through microscopic pores (termed ‘nanopores’) as a continuous strand, generating an electrical signal that is unique to each nt as it passes through the nanopore. Thousands of these nanopores are embedded in a synthetic membrane within a flow cell, allowing simultaneous sequencing of many individual molecules. Different flow cells have been developed to handle either DNA or RNA molecules, and some flow cell models contain a higher number of pores which increases the sequencing power at a higher financial cost. A computer then converts the electric signal into the corresponding nt. The maximum length of a nucleic acid sequence that can be analysed is limited to the length of the input material, with no theoretical upper limit. Read lengths up to 1 million bp have been reported [1], contrasting with the 100–400 bp length that is typical for short-read technology such as Illumina. Importantly, ONT technology continues to advance, and readers are advised to seek the most up-to-date information when planning experiments.

### Within the ONT platform, there are experimental options at the pre-analytic processing, library preparation and sequencing steps that influence the yield and cost of a sequencing experiment

At the pre-analytic processing stage, enrichment strategies such as PCR can be used to amplify sequences of interest within the sample, particularly for specimen types that have a relatively low abundance of the sequence of interest compared with host DNA or environmental micro-organisms. A higher abundance of the target sequence typically leads to higher-quality sequencing results and may also extend the usability of flow cells by reducing unnecessary, non-specific sequences from exhausting the nanopores.

Library preparation for ONT offers ways to customize and optimize experimental approaches. The attachment of an ONT-specific molecular adapter is necessary to allow the nucleic acid sequences to move through the nanopores. ONT offers several chemistry kits to accomplish this task, either by using transposons to insert the adaptors into the middle of the sequence or by ligating adaptors to the ends of the sequence. Currently, the transposon-based approach requires fewer reagents and is a faster workflow, making it potentially more useful in LMICs. Samples can be barcoded and run in batches of up to 96 individual samples using ONT chemistry kits. Supply chain, storage requirements, third-party reagents and experimental goals are all considerations when selecting the ONT chemistry kit for an experiment.

The sequencing portion of the workflow requires both a reusable sequencing device and a consumable flow cell component, both of which offer flexibility in sequencing capacity and portability. The MinION Mk1B (also referred to simply as the ‘MinION’) is a commonly used portable device that requires a connection to a separate computer to perform sequencing. Laptops used with the MinION Mk1B must contain enough computational power to perform the sequencing. The GridION sequencer and other integrated benchtop-sized devices can process multiple flow cells at once. The PromethION sequencer uses higher capacity flow cells to enable higher-coverage sequencing and is available as modular or fully integrated devices. The ability to access computationally sufficient computers and the internet may impact the experimental approach if researchers cannot access these tools.

The selection of the flow cell model depends on the sequencing machine and experimental goals. The MinION flow cell is a commonly used model. Flow cells can be re-used after a sequencing run via a washing procedure that removes the prior specimen and re-sets the nanopores, though the number of available sequencing pores tends to decrease over time with each flow cell use, and flow cells cannot be re-used indefinitely. Flow cell design has improved over time with improved accuracy, with the most recent MinION flow cell (model 10.4.1) reported at >99% accuracy [2], much improved from the initial 15% error rate when nanopore technology was first developed.

During sequencing, nucleic acids are read in real time, which offers unique opportunities. Sequences can be mapped to existing reference genomes while sequencing is ongoing, enabling researchers to potentially shorten the duration of sequencing by stopping the experiment when adequate results are achieved. It is also possible to enrich target sequences through ‘adaptive sampling’ during live sequencing, whereby a computer monitors the nanopores for specific sequences of interest as they pass through the nanopores in real time. Adaptive sampling is unique to ONT compared with other platforms and may reduce the need for chemically based enrichment approaches and their associated reagent costs but requires advanced bioinformatic knowledge and adequate computational power.

### Nanopore sequencing has been used successfully for accurate, real-time surveillance of infectious disease outbreaks

Nanopore sequencing was used as part of the 2015 Ebola virus epidemic in West Africa for real-time genomic surveillance, generating results within 24 h after sample acquisition [3]. This work was done in connection with the ARTIC network, a collaboration between the University of Birmingham, the University of Edinburgh, the University of Cambridge and the Wellcome Trust, which aims to develop affordable, mobile viral sequencing systems that can be used in outbreak settings worldwide [4]. Subsequently, the severe acute respiratory syndrome coronavirus 2 (SARS-CoV-2) pandemic created an urgent need for rapid analysis of viral variants throughout the world. The ARTIC network developed a multiplex primer scheme and associated computer software for real-time analysis of SARS-CoV-2 on ONT platforms and, importantly, shared this approach with the international community to enable rapid data collection to monitor the evolving SARS-CoV-2 genome during the outbreak [5]. One study in Germany [6] adapted this approach into a simplified laboratory and bioinformatic workflow on clinical samples that were positive for SARS-CoV-2 on real-time PCR. They performed real-time basecalling to optimize the balance between data collection and flow cell usage. The workflow time from RNA extraction to whole-genome sequence result was 7 h for an individual specimen and 11 h for multiplexed specimens, and they were able to use each flow cell three times for an estimated cost of $40 USD per sample. Nanopore sequencing for SARS-CoV-2 whole-genome sequencing was found to detect single nt variants with >99% sensitivity when compared with the short-read sequencing platform Illumina as the gold standard [7]. These methods facilitated the generation of SARS-CoV-2 genomic surveillance data in many countries, but systematic efforts are needed in order to understand how ONT sequencing is used for pathogen surveillance in LMICs.

In many LMICs, clinical microbiology laboratories rely on culture-based methods and point-of-care testing to detect pathogens. However, these approaches may be insufficient depending on the clinical or research question. Culture-based bacterial and fungal identification requires the use of selective culture media, which limits the range of detectable organisms and may miss fastidious pathogens. NGS via metagenomic or targeted approaches can detect a wide variety of bacteria, viruses and fungi from a single clinical sample, including pathogens not identified on microbiological culture [8], and may help overcome this challenge. Second, phenotypic antimicrobial susceptibility testing via disc diffusion can help identify antimicrobial resistance (AMR) but requires consistent access to a range of antibiotic discs and provides limited insight into resistance mechanisms. It does not reveal specific resistance genes or allow for genomic epidemiology to assess transmission between contacts. Particularly when sample volume is limited, ONT can simultaneously detect pathogens and resistance genes in a single assay [8]. Conducting this analysis locally – without the need to export samples – can build laboratory capacity and help address global health inequities.

### Understanding how researchers in LMICs use ONT requires the evaluation of equity indicators in research publications

Inequities in authorship representation persist for authors from LMICs where the studies themselves are conducted. One study evaluated publications from 2014 to 2018 in PubMed that were conducted in LMICs and found that 14.8% demonstrated ‘authorship parasitism’, meaning that no authors were affiliated with the country where the study took place [9]. Another study reviewed authorship guidelines described by global health-focused journals (*N*=45) and found that only 17.8% (*N*=8) of these described criteria for inclusion of local authorship for research conducted in LMICs [10]. Of these, only three journals required the inclusion of authors who were affiliated with the study country. In evaluating the use of ONT worldwide, the inclusion of local scientists as leading authors can serve as an indicator of equitable use of this technology. Additionally, understanding if studies perform ONT sequencing in the same country where samples are collected will provide valuable insights into the equitable and practical use of ONT worldwide.

### Nanopore sequencing has the potential to expand laboratory capacity for molecular diagnostics in LMICs worldwide, but the practical use and implementation in Africa have not been evaluated

The authors recognize that the African continent is heterogeneous with a variety of countries, communities and healthcare settings with different levels of resource access. However, understanding the use of ONT technology in Africa to study human infectious diseases from technologic and equity standpoints can inform future research efforts within the field of molecular epidemiology in global settings. In this scoping review, we:

1. Describe the current use of ONT in Africa for surveillance and diagnosis of human infectious diseases in regard to common pathogens, study populations and laboratory approaches,
2. Discuss applications of ONT for real-time detection of infectious pathogens in Africa and
3. Identify challenges, existing gaps and opportunities for future research studies to design experiments utilizing ONT in Africa.

## Methods

### Study framework

This scoping review was conducted by using the framework for scoping review as described by the Joanna Briggs Institute (JBI) Reviewer’s Manual [11] in accordance with the Preferred Reporting Items for Systematic Reviews and Meta-analyses extension for scoping review (PRISMA-ScR) guidelines [12] and the JBI methodology for scoping reviews [13]. Prior to data collection, the protocol was made publicly available on Open Science Framework [14].

### Search strategy

**Fig. 2.**
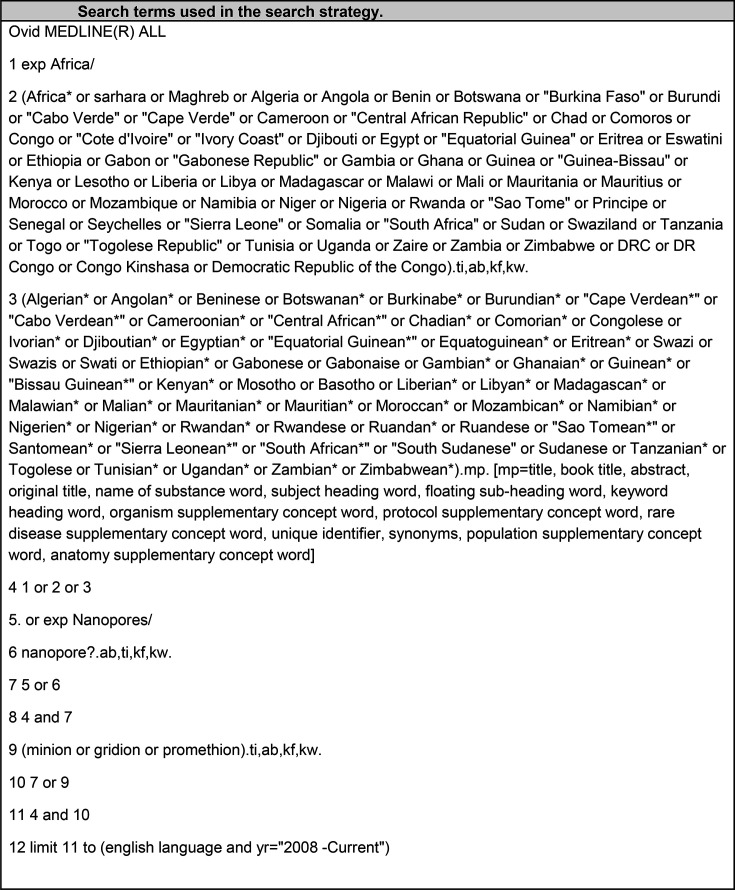
Search terms used in the search strategy.

The search strategy was developed by a health sciences librarian (B.O.) and utilized controlled vocabulary and natural language search terms to capture the concepts of nanopore, MinION, GridION or PromethION. The search hedge developed by Campbell was adapted to identify studies related to Africa [15]. The search was developed for MEDLINE(R) ALL (via Ovid) and then translated to the following databases: Embase+Embase Classic (via Ovid), Scopus, Web of Science Core Collection (SCI-Expanded, SSCI, AHCI, CPCI-S, BKCI-S, BKCI-SSH, ESCI, CCR-Expanded, IC) and African Index Medicus from their inception to 30 April 2023. Search results were limited to the English language and a publication date of 2008, but no other limits were applied. Despite the hundreds of languages spoken in Africa, one study found that ~98% of the active biomedical journals from Sub-Saharan African (SSA) countries are published in English [16], and therefore, our search criteria were expected to encompass the majority of published studies. This date range was chosen because ONT was trademarked in 2008, and it is not expected to find published results prior to this. The search string of the MEDLINE (Ovid) database is shown in Fig. 2. The included full-text articles were used to complete a manual search of the references as well as a forward citation search of all articles included in the study.

Included papers were limited to full-text pre-print and published studies of any study type including experimental studies such as randomized and non-randomized controlled trials; prospective and retrospective cohort studies, case-control studies, surveillance studies and cross-sectional studies; and descriptive study designs including letter to the editor, case reports, case series and cross-sectional studies. Sources such as grey literature, conference abstracts, thesis papers, text and opinion papers were excluded due to insufficient details available to answer the study questions. Studies with at least one specimen derived from humans of any age or gender (either cultured isolates or primary specimens) that included at least one microbial target were included. Studies that only utilized non-human specimens (animal, plant and environmental) were excluded. Studies examining samples that were obtained in Africa and sequenced elsewhere were included.

**Fig. 3.**
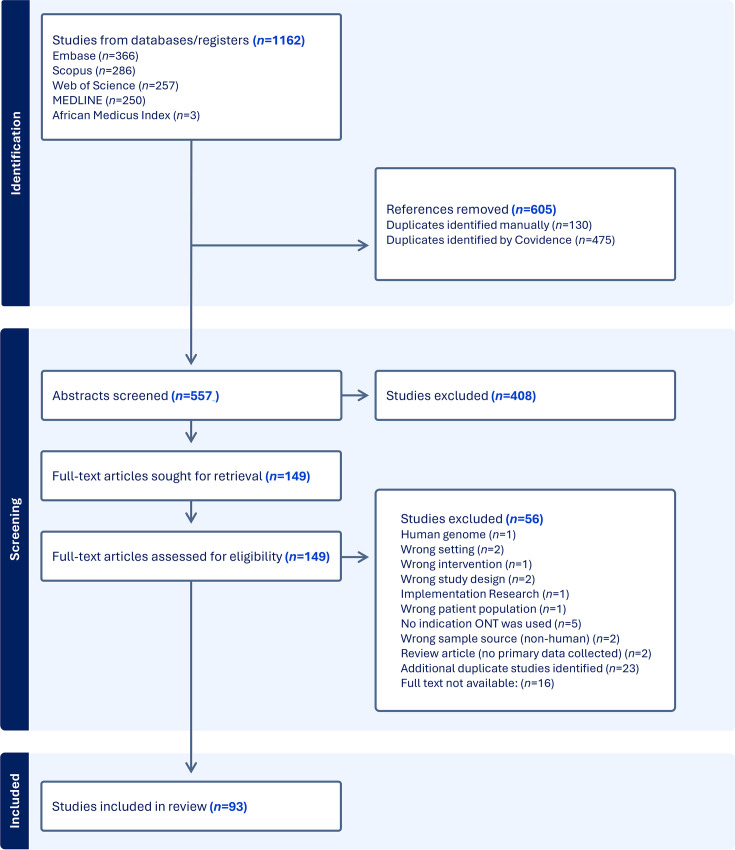
PRISMA flow chart diagram of the search strategy.

Identified citations were collated and uploaded into Covidence, and duplicates were both automatically and manually removed. Following a pilot test, titles and abstracts were screened by two independent reviewers (K.B. and U.U.) for assessment against the inclusion criteria for the review. Potentially relevant sources were retrieved in full, and their citation details were uploaded into Covidence. The full text of selected citations was assessed in detail against the inclusion and exclusion criteria by two independent reviewers (K.B. and U.U.). Reasons for the exclusion of sources that did not meet the inclusion criteria during full-text review were recorded and reported. Any duplicates, such as a pre-print and published paper from the same author and study, were manually excluded. Any disagreements between the reviewers at each stage of the selection process were resolved through discussion or with an additional third reviewer (B.T. and M.O.). The results of the search and the study inclusion process are presented in a PRISMA-ScR flow diagram (Fig. 3).

### Data extraction and analysis

Data was extracted from papers that met inclusion criteria by two independent reviewers (K.B. and U.U.) using a data extraction tool developed by the reviewers in REDCap. The data extracted included specific details about the author order and affiliation, study, participants, concept, context, study methods and key findings relevant to the review questions (Fig. S1, available in the online Supplementary Material). If a study referenced a previously existing protocol or other study for full methodology, that source was accessed if it was required to answer the study questions. Data was exported from REDCap into Microsoft Excel for analysis. Descriptive statistics such as counts and per cents were used to analyse the data.

## Results

The database search generated 1,162 results. After the removal of duplicates and the application of inclusion and exclusion criteria, 93 studies were included in this review (Fig. 3). Four studies were preprints at the time the search was conducted (three preprints in 2023 and one in 2024).

### The use of nanopore technology in Africa for surveillance and diagnosis of human infectious diseases

#### Authorship and conflicts of interest

**Fig. 4.**
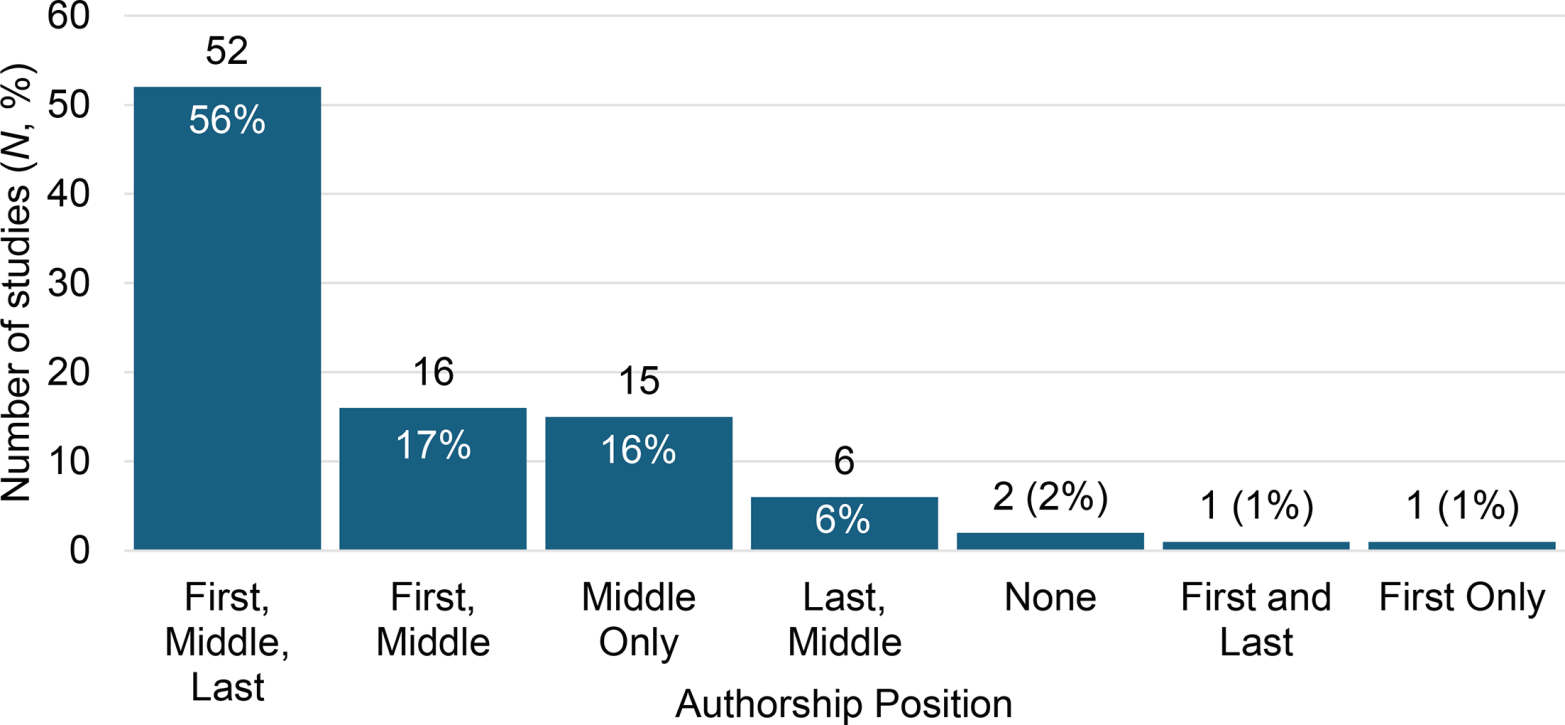
African-affiliated authors present in different authorship positions amongst studies.

Thirty-one studies included multiple co-first authors, and 21 studies included multiple co-last authors. Fourteen studies (15%) were composed of only authors who were affiliated with African institutions, while two studies (2%) did not include an African-affiliated author. The most common authorship team structure included having an author affiliated with an African institution (termed ‘African-affiliated’) in all three positions of the paper, i.e. as first, middle and last authors (*N*=52, 56%, Fig. 4).

#### Specimen location

**Fig. 5.**
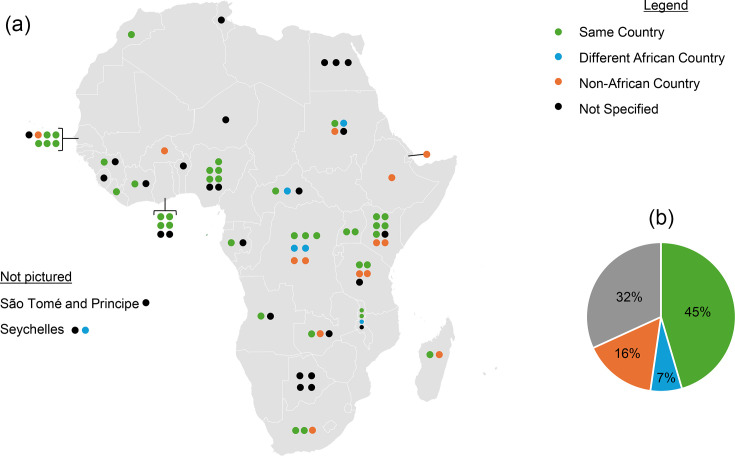
(a) Location of ONT sequencing in studies that analysed specimens from a single country (*N*=88)*. Circles denote one study each and are located within the country where specimens were collected and not geographically representative of the location. The circle colour corresponds to the location of sequencing. (b) The pie chart indicates the sequencing location across the 88 studies. *Angola (2), Benin (1), Botswana (4), Burkina Faso (1), Central African Republic (3), Côte d'Ivoire (2), Djibouti (1), the DRC (7), Egypt (3), Ethiopia (1), Gabon (2), Ghana (6), Guinea (2), Kenya (8), Liberia (1), Madagascar (2), Malawi (3), Morocco (1), Niger (1), Nigeria (7), São Tomé and Príncipe (1), Senegal (7), Seychelles (2), Sierra Leone (1), South Africa (3), Sudan (4), Tanzania (5), Tunisia (1), Uganda (2) and Zambia (3). Five studies are not pictured due to sample collection from multiple African countries. These included (1) Burkina Faso and Ethiopia; (2) Burkina Faso, Niger and Nigeria; (3) Cameroon, Niger and Nigeria; (4) Algeria, Cameroon, Niger, Senegal and Tunisia; and (5) Madagascar, South Africa and England. (Gray base map created in Microsoft Excel, Powered by Bing, © GeoNames, Microsoft, OpenStreetMap, TomTom)

Eighty-eight studies analysed specimens from a single African country (Fig. 5). Of these, five countries accounted for nearly 40% (*N*=35) of all publications including Kenya (*N*=8), Nigeria (*N*=7), the Democratic Republic of the Congo (DRC, *N*=7), Senegal (*N*=7) and Ghana (*N*=6). Five studies analysed specimens from multiple African countries, for a total of ten different countries within those five studies [for the five studies that included samples from more than one country, sample locations were the following: (1) Burkina Faso and Ethiopia, (2) Burkina Faso, Niger, Nigeria, (3) Cameroon, Niger, Nigeria, (4) Algeria, Cameroon, Niger, Senegal, Tunisia (5) Madagascar, South Africa, and England]. The location where ONT sequencing occurred was clearly specified in 65/93 studies (70%). Four studies performed ONT sequencing in more than one location, and one of these four studies collected samples from >1 African country.

#### Sequencing location

For studies that analysed specimens from a singular location (*N*=88), the sequencing location was clearly discernible in 60/88 (68%) of studies. For these studies, the sequencing location was in the same country of specimen collection for 40 studies (45%), a non-African country in 14 studies (16%), a different African country in 6 studies (7%) and unspecified in 28 studies (32%) (Fig. S2). For all studies, 20 African countries were identified that performed ONT sequencing in-country, whether for samples collected in that country itself or a different country. The top six countries by publication number were Kenya (8), Nigeria (6), Ghana (5), Senegal (5), the DRC (4) and South Africa (4) (Fig. S3), which were responsible for 32 of the 49 instances where ONT was performed in Africa (65%). Two studies utilized a private company outside of Africa for ONT sequencing, and for both of these studies, the details of the sequencing protocol were not publicly accessible. Of the studies that performed sequencing in a non-African country (*N*=16), the most common location was England (*N*=4, 16%) followed by Norway (*N*=3), Germany (*N*=2), the USA (*N*=2), Belgium (*N*=2), China (*N*=1), France (*N*=1) and Japan (*N*=1).

#### Study characteristics

Table 1 summarizes the studies in regard to patient population, number of specimens and types of specimens analysed. The majority of studies examined specimens with de-identified patient data. However, children’s samples were included in about one-third of studies, and a small number of studies utilized a One Health approach and incorporated either animal or environmental samples in addition to human specimens. Some studies utilized ONT to analyse a subset of their total study specimens, but the majority of studies (*N*=91/93) clearly reported the number analysed by ONT. This was typically a small or medium-sized number of samples (*N*=31 and *N*=40, respectively). Patient specimens were directly analysed in about two-thirds of studies, the most common of which was blood, serum or plasma (*N*=34).

**Table 1.** Study populations and specimen types

Study characteristic	Reported in study*N* (%)	Comment
**Study population**		
Age	35 (38)	
Children (age <18)	31 (33)	ONT clearly used to sequence children’s specimens in 28/31 studies
Persons living with human immunodeficiency virus (PLWH)	5 (5)	ONT clearly used to sequence PLWH’s specimens in 4/5 studies
Pregnant people	1 (1)	
One Health	4 (4)	ONT used on a human sample PLUS an environmental sample OR animal sample
**Study size**	91 (98)	
<10	31 (33)	
11–99	40 (43)	
>100	18 (19)	
>1,000	2 (2)	
**Specimen type**	93 (100)	Direct patient specimen 58/93 (62%) Cultured isolate 33/93 (35%) Both direct specimen and cultured isolate 2/93
Blood, serum or plasma	34	Some studies tested multiple sample types; therefore, results do not reflect an additive total
Nasopharyngeal or oropharyngeal	25	
Stool	12	
Urine	9	
Cerebrospinal fluid	6	
Skin and soft tissue	6	
Lower respiratory tract	4	
Vaginal	3	
Dried blood spot	2	

### Surveillance and diagnosis

#### Clinical applications of ONT

Only one study demonstrated the use of ONT in a clinical setting to inform care for a patient [16]. The authors were called to travel to a hospital with the MinION device and used a portable, non-targeted metagenomic approach to analyse a stool sample of a hospitalized, clinically ill child with dehydration and loose stools. They identified the presence of *Campylobacter jejuni* in the child’s stool, a finding that resulted in the prescription of erythromycin for the patient. While other studies examined clinical samples, the results were not clearly linked to changes in patient care or outcomes. Furthermore, it was not always clear if the patient specimens were recently collected or retrieved from a storage facility, limiting the ability to draw conclusions.

#### ONT utilization during the SARS-CoV-2 pandemic

Twenty-five total studies (27%) used ONT to study the SARS-CoV-2 virus. None of the SARS-CoV-2-related publications discussed any aspect of their workflow timeline (i.e. time from specimen acquisition to result), so it is not known if the results were used to inform public health decisions in real time. The Illumina sequencing device was used in addition to ONT in 5/25 (20%) of SARS-CoV-2 studies. Two studies examined an additional pathogen alongside SARS-CoV-2 (influenza virus in one study and other human coronaviruses in one study). Of the 25 publications, 1 study included specimens from Senegal, Niger, Tunisia, Cameroon and Algeria, and the other 24 studies examined specimens from a single country.

#### Pathogen detection and surveillance

**Fig. 6.**
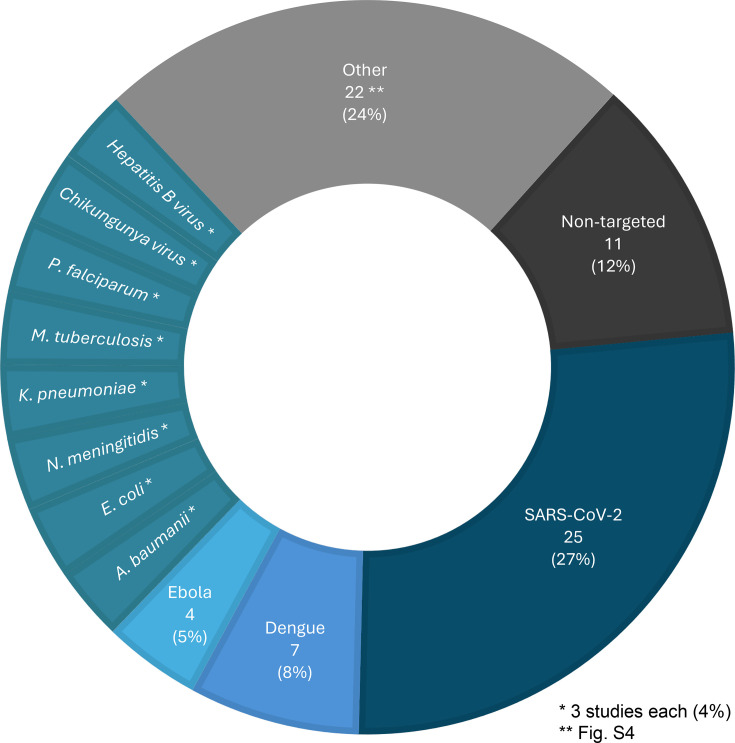
Pathogens detected in studies, *N* (%). Eleven studies utilized a non-targeted metagenomic approach, whereas the remainder developed assays targeted towards specific pathogens of interest.

Other than the SARS-CoV-2 virus, the next most common pathogens studied were dengue virus (*N*=7), Ebola virus (*N*=4) and three studies each for *Acinetobacter baumannii*, *Neisseria meningitidis*, *Escherichia coli*, *Klebsiella pneumoniae*, *Mycobacterium tuberculosis*, *Plasmodium falciparum*, chikungunya virus and hepatitis B virus (Figs 6 and S4). Nineteen studies used ONT during an infectious disease outbreak other than SARS-CoV-2 including (in alphabetical order) chikungunya virus, dengue virus, Ebola virus, Lassa virus, *P. falciparum*, monkeypox virus, *N. meningitidis*, poliovirus and Sudan virus (Fig. S5). Four of these clearly reported obtaining results in real time.

### ONT for real-time detection of infectious diseases in Africa

#### Duration of time required to achieve results

A total of ten studies reported at least one additional aspect of the workflow timeline other than sequencing duration. Four studies reported the time from specimen acquisition to result with a time ranging from less than 2 days to 10 days. Four studies reported the time from nucleic acid extraction to result, ranging from 14 h to 30 days. Five studies reported that a result was obtained in <7 days from the time of specimen collection. Two studies reported multiple aspects of the workflow duration, with one reporting 10 days from sample acquisition to result, with 6 h from library preparation to result. The other reported 1 day from both sample acquisition to result and nucleic acid extraction to result.

#### Sequencing workflow

The ability to detect infectious diseases in real time depends on the sequencing workflow that can be accomplished on-site, which is dependent on access to equipment and reagents for any supplementary workflows that are needed. Nearly all studies (97%) used a commercially available DNA or RNA extraction kit (Table 2). We found that 90% of studies first used a non-ONT confirmatory method to establish the presence of the pathogen of interest, prior to using ONT. The majority of studies used PCR to enrich a clinical sample for a pathogen prior to using ONT. The use of barcoding to multiplex multiple samples into a single run was reported in 71% of studies and was most commonly performed using ONT-specific barcoding kits. Sixty per cent of studies reported the use of a flow cell, which is a required component of ONT sequencing. Less than one-third reported the duration of sequencing, and only 3/93 studies reported the number of times each flow cell was used. The use of negative controls during sequencing was clearly reported in only 12% of studies. Whole-genome sequencing was the study goal in 3/4 of studies, and a smaller number examined AMR genes (28%) or specifically plasmid-related genes (18%).

**Table 2.** Study results pertaining to a typical NGS workflow

Study characteristic	Reported*N*/total *N* (%)	Comment
**Nucleic acid extraction**		
Commercially available kit	90/93 (97)	
Custom extraction method	3/93 (3)	(1) Alkaline lysis, (2) salting out, (3) chloroform extraction
**Pre-analytic processing**		
PCR utilized	57/93 (61)	39/57 (68%) utilized tiled amplicon PCR 2/57 studies used the ONT 16S Rapid Barcoding Kit
Confirmatory method used prior to ONT	84/93 (90)	Methods included PCR, serologic positivity, previously identified microbial culture and rapid antigen positivity
Hybrid capture or adaptive sampling	0/93 (0)	
**Barcoding**	66/93 (71)	66 studies reported using barcoding of any type; no studies explicitly stated it was not used
ONT Native Barcoding Kit	26/66 (39)	
ONT Rapid Barcoding Kit	28/66 (42)	
ONT 16S Rapid Barcoding Kit	2/66 (3)	
Other commercial kit	6/66 (9)	
Custom approach	1/66 (2)	Nested PCR primers targeting major capsid protein VP1
Approach not specified	3/66 (5)	
Medium-sized studies (11–99 samples)	33/40	studies described the use of barcoding
Large-sized studies (100–999 samples)	7/18	studies described the use of barcoding
Very-large sized studies (>1,000 samples)	1/2	studies described the use of barcoding
**Flow cells**	56/93 (60)	All studies utilized MinION flow cells, most commonly version 9.4 (*N*=34) or 10.4 (*N*=3)
Number of samples analysed on a single flow cell (for studies that examined >1 specimen)	19/84 (23)	Ranged from 4 to 96 samples per flow cell
Duration of flow cell use (sequencing duration)	26/93 (28)	3 min to 48 h
Number of uses per individual flow cell	3/93 (3)	Two studies stated flow cells were not re-used; one study used each flow cell two times
**Negative control**	11/93 (12)	
**ONT sequencing machine**	91/93 (98)	
MinION Mk1B	60/91 (66)	2/60 studies also described the use of an external computer (a required component for this device)
GridION	20/91 (22)	Benchtop device can run 5 MinION flow cells concurrently
MinION Mk1C	5/91 (5)	This portable model contains a built-in computer
2 or more ONT devices	6/91 (7)	
ONT alone (no other sequencing device)	49/91 (54)	
ONT+Illumina	38/91 (42)	
Sanger	4/91 (4)	
PacBio	1/91 (1)	
**Data accessibility**		
GitHub utilized	45/93 (48)	Authors reported utilizing GitHub to disseminate information
Any sequence uploaded to a public database	70/93 (75)	Most common databases were the Global Initiative on Sharing All Influenza Data (GISAID, *N*=30 studies), GenBank (*N*=26 studies) and the European Nucleotide Archive (*N*=16 studies); some studies uploaded more than one sequence to more than one database, so results are not additive
Whole-genome sequence uploaded to a public database	55/93 (59)	
**Results**		Some studies examined a combination of result types, so results are not additive
Whole-genome sequencing	71/93 (76)	38/71 (54%) reported the per cent of genome coverage achieved
Antimicrobial or antiviral resistance genes	26/93 (28)	
Plasmid	17/93 (18)	

### Challenges and opportunities

#### Financial cost of the ONT workflow

The minority of authors (14/93, 15%) discussed financial cost as a potential benefit of using ONT in their abstract, introduction or discussion. Five total studies reported the per-sample cost of their study results. A study by Shaw *et al.* [17] in the DRC compared nanopore sequencing for poliovirus detection in stool samples to the gold standard, cell culture-based detection. They reported a per-sample cost of $20 USD per sample for multiplexed runs with 96 samples on an ONT flow cell compared with $31 USD per sample for the culture-based approach. Another study by de Cesare *et al.* [18] used nanopore sequencing for the detection of malaria on dried blood spots in Zambia and reported a cost of $25 USD per sample when multiplexing 96 samples together on a flow cell and an additional $1 USD/sample cost if Flongle flow cells were used to sequence smaller batches of 24 samples. They reported that a custom enrichment approach using reduced volume and selective whole-genome amplification (sWGA) saved $4 USD per sample compared with standard volume sWGA. Another study by Girgis *et al.* [19] used ONT to analyse dried blood spots *for P. falciparum* resistance genes in Ghana and calculated a per-sample cost of $35 USD. One study available as a pre-print at the time of this study by Tshiabuila *et al.* (now available as a full publication [20]) developed an ONT assay for whole-genome sequencing of hepatitis B virus and compared it to an existing Illumina assay. They found that the USD cost per sample was $10–40 with ONT and $150–250 for Illumina. A slightly older study by Tafess *et al.* [21] investigated antibiotic resistance genes in *M. tuberculosis* isolates with the MinION compared with MiSeq. The MinION samples were multiplexed into 12 samples per run with a per-sample USD cost of $71.56, while the MiSeq samples were multiplexed in batches of 24 with a per-sample cost of $67.83.

Overall, we identified a need for improved reporting of ONT methodology including pipeline timelines, cost, use of barcoding, flow cell models and the use of negative controls (Table 3). Publications that provide these details will enhance reproducibility and support the development of new studies using ONT for the diagnosis and surveillance of infectious diseases in low-resource settings.

**Table 3.** Current gaps and proposed solutions for research studies using ONT in Africa and other LMICs

Gap	Justification	Solution
**Timely results**	Workflow time discussed in 10.7% of studies	Clear reporting for standardized steps of the sequencing workflow
**Cost**	Sample cost discussed in 5.4% of studies	Authors report the estimated per-sample cost
**Reproducibility of ONT-specific methodology**	ONT technology is rapidly evolving, and increased methodologic transparency is needed in publications to enhance reproducibility: Barcoding not described by 25% of studies with >11 specimens Flow cell model not specified by 40% of studies Flow cell re-use described in 3.2% of studies 12% of studies clearly reported the use of a negative control during sequencing	Detailed description of nanopore-specific study design in the ‘Methods’ section Protocols are posted in publicly accessible repository (i.e. protocols.io) Peer review process includes reviewers with expertise in nanopore sequencing
**Sequence generation**	Sequencing location was not clearly discernible in 30% of studies	Clearly describing sequencing location will improve understanding of ONT use in LMICs
	Genomic data was uploaded to public databases in 75% of studies	Authors should clearly describe methodology for uploading data into public databases such as GenBank, SRA, ENA or GISAID
**Clinical utility**	One study clearly used ONT to directly influence patient care	More studies using ONT for clinical applications are urgently needed in LMICs

ENA, European Nucleotide Archive; GISAID, Global Initiative on Sharing All Influenza Data; SRA, Sequence Read Archive.

## Discussion

This is the first scoping review in the published literature that describes the use of ONT to detect human infectious diseases in Africa. This report offers new insights into several aspects of the practical adoption of ONT into LMICs in recent years.

### ONT sequencing is utilized by African scientists

This study identified authorship team structures that included African-affiliated authors in all three authorship positions (first, middle and last) in the majority of studies. Additionally, 15% of all studies were completed by an entirely African-affiliated authorship team. This is encouraging and suggests that the use of ONT in Africa does not depend on non-African partners to bring in reagents or technology from elsewhere, though this is not a proven assumption and is a topic for future investigation. In this review, only 2% of studies displayed ‘authorship parasitism’, which is smaller than a previously reported 14.8% author parasitism rate [9]. Evaluating the country of submission for genome sequences generated with ONT may represent another way to evaluate the equitable use of ONT technology but was beyond the scope of this study. A previous study [22] examined metadata from GenBank and found that nearly 75% of all sequences were uploaded by the global north, with 70% originating from three countries, the USA, China and Switzerland. Sequences deposited into GenBank by locations in the global south contained long reads more frequently (25.7%) than those in the global north (20.2%), suggesting that long-read sequencing such as ONT may be used more frequently in certain parts of the world.

### ONT sequencing is used in-country to sequence specimens collected from people living in Africa

Sequencing was performed in the same country where specimens were collected in almost half of all studies, while about one-third of studies did not provide a clear enough description to deduce where ONT sequencing occurred. The only ONT sequencers used were the MinION and GridION, suggesting that the more powerful PromethION has not become widespread. ONT sequencing was used alone in about half of the studies, supporting that access to ONT alone rather than any other sequencers (Illumina, PacBio) can generate publishable sequencing results. The recent SARS-CoV-2 pandemic may have influenced country-specific and regional genomic sequencing capacity by incorporating ONT sequencing into existing laboratories. In 2021, the World Bank conducted a landscape analysis [23] of global networks that supported genomic surveillance and determined that the SARS-CoV-2 pandemic resulted in strengthened genomic surveillance networks in Africa, including the establishment of 12 SARS-CoV-2-specialized laboratories in partnership with Africa CDC located in Senegal, Ghana, Nigeria, the DRC, South Africa and Uganda. Other genomic hubs were in Gambia, Mali and the DRC. In our study, ONT sequencing occurred in all of these locations except for Mali and Gambia. However, the same report also identified inadequate surveillance capacity and heterogeneity in the region defined as SSA. Eastern and Southern SSA had higher sequencing capacity, while Central Africa had significant gaps in surveillance facilities. Some African countries had no genomic sequencing capacity, including Liberia, Namibia, South Sudan, Mauritania, Seychelles and Tanzania. We identified one study in which specimens were both collected and analysed in Liberia. Specimens from Seychelles were included in one study and sequenced in Kenya, while specimens from Tanzania were included in five studies with two conducting sequencing in-country, two in a non-African country and one with a sequencing location not discernible. Namibia, South Sudan and Mauritania did not perform any sequencing, nor were specimens from the countries included in published studies. The World Bank Report [23] found that 70% of sequencing capacity was situated outside of the National Public Health Institutes, and 38% of all sequencing devices in Africa were located in South Africa. Our study revealed that 4.3% of studies performed sequencing in South Africa, suggesting that ONT technology may be more evenly distributed than other types of NGS technology.

### The broad range of pathogens, whole-genome sequencing during infectious disease outbreaks and ability to detect AMR genes reveal a broad use of applications for ONT for pathogen surveillance in LMICs

Most studies targeted a specific pathogen of interest rather than a non-targeted approach. The most common pathogen studied was SARS-CoV-2, which is consistent with the increased utilization of the portable MinION sequencer during the global SARS-CoV-2 pandemic. A broad range of pathogens and specimen types were identified, including bacteria, RNA and DNA viruses, the parasite *P. falciparum*, the fungi *Cryptococcus neoformans* and *Cryptococcus gattii* and several mycobacterial species. For the purpose of this review, surveillance activities included both surveillance for pathogens or AMR genes and encompassed the majority of ONT applications (77% of all studies). A total of 19 studies used ONT during non-SARS-CoV-2 infectious disease outbreaks, though only 4 clearly reported generating real-time results. In all of the outbreak-related studies, ONT was not used to diagnose patients with the illness, but rather was used for genomic surveillance after a confirmatory test first established the diagnosis in a patient. Generating a full list of AMR genes that were examined was beyond the scope of this paper, but examination of AMR genes was a common application that was done in almost one-third of all studies.

### ONT sequencing was not a primary method for the clinical diagnosis of infectious diseases in humans in Africa but shows promise for clinical applications

ONT has been used for clinical diagnosis in other settings in high-income countries, including a real-time metagenomic-based approach for diagnosis of lower respiratory tract infections in hospitalized patients [8] with a median turnaround time of 6.7 h and prospective detection of pathogens in bile duct fluid with results obtained within 8 h [24]. Studies using ONT in Africa continue to emerge, with one study in Eritrea using the MinION to detect drug-resistant tuberculosis in clinical samples with a turnaround time of 5–6 h [25], and another study in Ghana using ONT to study the genetic diversity, AMR genes and virulence of *Vibrio cholerae* in clinical and environmental samples [26]. In our study, a single case report was identified that used ONT for a clinical application [16]. The authors used a non-targeted sequencing approach to analyse the stool of a child with diarrhoea and dehydration, which generated metagenomic data on the entirety of the organisms and host DNA detected in the child’s stool. Interpreting metagenomics results such as this in a clinical setting can be challenging due to a lack of gold standard criteria to discern colonizing organisms from true pathogens, but the results of this test led to the prescription of erythromycin to target * C. jejuni* and resulted in clinical improvement.

Clinical diagnosis from direct patient specimens may be challenging due to the need for enrichment strategies to enhance pathogens that are present in low abundance. The majority of studies in this review used pre-analytic processing to enrich samples for pathogen DNA or confirm the diagnosis of a specific pathogen prior to sequencing. Enrichment strategies may add to the per-sample cost, which is important when considering clinical utility and the ability to scale ONT for widespread use in LMICs. However, the per-sample cost was very under-reported in the papers identified in this review, reported in only five studies. Three of these compared ONT with Illumina workflows and found that ONT had a lower per-specimen cost, which supports that ONT can aid in diagnostic testing in resource-constrained areas. A unique feature of ONT sequencing is the ability to perform adaptive sampling, which can enrich pathogens without additional reagents, but this approach requires competency in bioinformatics. The ONT bioinformatic analysis program ‘Epi2Me’ is a user-friendly application, but it requires either a stable and fast internet connection to analyse sequence data online, or a powerful laptop computer that can download and run the software offline. Custom bioinformatic pipelines can be designed to run off-line, but these require additional bioinformatic skills to create and implement. Notably, all sequencing runs require internet access in order to initiate the sequencing, which may create a barrier in some settings, but a sustained internet connection is not necessary. No studies used adaptive sampling, and the cost savings from this approach are not known. Developing approaches that incorporate this technology into patient testing may contribute to decreased per-sample costs, but additional research is needed.

Finally, the turnaround time from sample collection to result greatly influences clinical utility. In some centres, traditional culture-based methods may take days to weeks for results to become available, which is not a clinically actionable timeframe. This review identified several studies that obtained results within 1–2 days, which demonstrates that ONT results can generate clinically useful information. Overall, the lower cost and fast turnaround time of ONT sequencing shows promise for clinical diagnostics in LMICs, including for infectious pathogens and host genetic syndromes to inform point-of-care decision-making. Additional work is needed to develop pipelines for clinical diagnostics in these settings.

### Reproducibility of ONT-based workflows will be enhanced by increased reporting of laboratory approaches in the following topic areas

#### Flow cell usage

The use of the specific model of flow cell was not described in 40% of studies. As ONT technology advances, updated flow cell technology has demonstrated successive improvements in accuracy from prior versions, and therefore, including the specific flow cell version in publications is crucial. Flow cell washing and re-use is an important metric given that sequencing depth may be compromised as the number of uses increases due to lower availability of sequencing pores, as well as the potential to decrease cost if flow cells are re-used multiple times. The number of times flow cells were used was only specified in 3/93 studies.

#### Multiplexing

Multiplexing samples through the use of barcoding is an important aspect of any study using NGS, particularly due to the ability to decrease the per-sample cost. In studies with at least a sample size of 11 or greater, 25% did not specify the use of barcoding. It is possible that some of these studies used barcoding without describing it specifically, but additional clarity will facilitate reproducibility.

#### Paired computer technology

The most commonly used device, the MinION sequencer, requires a connection to a laptop with sufficient computational power to perform sequencing. However, only two studies that used the MinION specifically described the use of a laptop. While not a primary aim of this study, four papers discussed that internet access was a barrier to the implementation of ONT technology.

#### Web-based links for methodologic reporting

Two studies used private companies located outside of Africa to perform the sequencing, and the authors of these papers cited the company website for methodology. In one case, the link the authors provided did not function. On the other, the authors provided a link to the company’s GitHub for the bioinformatic methods, but there was no link to the wet-lab methodology. These examples highlight the importance of ensuring methodological transparency to enable accurate interpretation of results and reproducibility.

### Limitations

This study reviewed only published studies, as grey literature or published abstracts were not expected to contain the level of detail required for data extraction required for this review. It is possible that more scientists are using ONT technology without resulting in publications, particularly due to the high financial costs associated with publication [27], and therefore, the use of ONT may be under-reported in published literature. While the majority of published literature from SSA is published in English [28], future studies could incorporate French language studies or quantify the number of non-English language publications that would otherwise meet study criteria. Additionally, an important step of ONT sequencing is the use of bioinformatics for data analysis, but evaluating the use of different bioinformatic tools was beyond the scope of this study due to the large number of available options, customization and heterogeneity of study needs.

## Conclusion

The potential for ONT to enable timely, low-cost and accurate sequencing in African countries is possible as demonstrated through a small number of studies that accomplished these goals individually, but more work is needed to improve the transparency and reproducibility of ONT applications in Africa. ONT was not primarily used for clinical diagnosis of infectious diseases. Most studies used ONT for genomic surveillance of pathogens or AMR genes and used a PCR-based approach to amplify a nucleic acid sequence of interest prior to ONT sequencing. The use of ONT to generate results with a short interval between specimen collection and result was described in a small number of studies. There is a need for improved reporting of ONT methodology including pipeline timelines, cost, use of barcoding, flow cell models and the use of negative controls. Publications that provide these details will enhance reproducibility and support the development of new studies using ONT for the diagnosis and surveillance of infectious diseases in low-resource settings.

## Supplementary material

10.1099/acmi.0.001020.v3 Uncited Supplementary Material 1.

## Abbreviations

AMR: antimicrobial resistance
DRC: Democratic Republic of the Congo
JBI: Joanna Briggs Institute
LMICs: low- and middle-income countries
NGS: next-generation sequencing
ONT: Oxford Nanopore Technology
PCR: polymerase chain reaction
PRISMA-ScR: Preferred Reporting Items for Systematic Reviews and Meta-analyses extension for scoping review
SARS-CoV-2: severe acute respiratory syndrome coronavirus 2
SR: scoping review
SSA: Sub-Saharan Africa
sWGA: selective whole-genome amplification
