# Supplementary material for: Utilization of Oxford Nanopore Technology for human infectious disease detection and surveillance in Africa: a scoping review
Source: Access Microbiol. 2025 Jul 15;7(7):001020.v3. doi: 10.1099/acmi.0.001020.v3 (PMC12263285; doi:10.1099/acmi.0.001020.v3)
Supplement: Uncited Supplementary Material 1. [file acmi-7-01020-s001.pdf]

Supplementary Figure 1: Detailed data extraction approaches	
Publication Information	Title, year, author names, author affiliations, author order, co-authorship, involvement by Oxford Nanopore Technology (funding, employees, investigative donations), journal
Participant Information	Age, involvement of special populations (children, pregnant women, persons living with HIV), total number, number sequenced via ONT
Study Approach	Specific pathogen of focus, plasmid sequencing, antimicrobial resistance gene, whole genome sequencing, location of sequencing, purposes for outbreak or surveillance activity, direct use in clinical care
Specimen Information	Organism (human required; any additional animal or environmental samples), total number of specimens in study, number of samples selected for sequencing via ONT and the number that were successfully sequenced, type of specimen (direct human specimen or cultured isolate), selection of sample for ONT based on previous lab study (i.e, known PCR-positive, serology positive, positive amplicons seen on gel), type of specimen (body fluid or source)
Specimen preparation	Reverse transcription approach if applicable, nucleic acid extraction approach, library preparation approach, barcoding kit, the use of PCR enrichment prior to ONT, the use of other specific enrichment techniques (hybrid capture probe, tiled amplicon PCR, 16s or 28 s PCR), the use of flow cells (model type, number of uses, number of washes), the use of DNA fragmentation
Sequencing Device	ONT device (flongle, MinION Mk1B, Mk1C, Gridion, Promethion), use of an additional sequence device (Illumina, Sanger, IonTorren, PacBio)
Basecalling and Bioinformatics	Length of sequencing time, link to a GitHub, use of external computer
Results	Time from sample acquisition to analysis or any steps within the NGS workflow, report of use of negative and positive controls, upload of genomic data to public database, result used clinically for a patient, discussion of cost by authors (in introduction and/or results)

Supplementary Figure 2: Location of sequencing for studies that examined specimens collected in a single country (N=88 total). The country of specimen collection is shown in the first column in alphabetical order.

Country	Total Number of Studies	Sequencing Location			
		Same Country	Other African Country	Non-African Country	Not specified
Angola	2	1	0	0	1
Benin	1	0	0	0	1
Botswana	4	0	0	0	4
Burkina Faso	1	0	0	1	0
Central African Republic	3	1	1	0	1
Cote d'Ivoire	2	1	0	0	1
Djibouti	1	0	0	1	0
Democratic Republic of the Congo	7	3	2	2	0
Egypt	3	0	0	0	3
Ethiopia	1	0	0	1	0
Gabon	2	1	0	0	1
Ghana	6	4	0	0	2
Guinea	2	1	0	0	1
Kenya	8	5	0	2	1
Liberia	1	1	0	0	0
Madagascar	2	1*	0	1	0
Malawi	4	2	1	0	1
Morocco	1	1	0	0	0
Niger	1	0	0	0	1
Nigeria	7	5	0	0	2
Sao Tome and Principe	1	0	0	0	1
Senegal	7	5	0	1	1
Seychelles	2	0	1	0	1
Sierra Leone	1	0	0	0	1
South Africa	3	2	0	1	0
Sudan	4	1	1	1	1
Tanzania	5	2	0	2	1
Tunisia	1	0	0	0	1
Uganda	2	2	0	0	0
Zambia	3	1	0	1	1
**Total**	**88**	**40**	**6**	**14**	**28**

*Sequencing occurred in both Madagascar and India but is counted once as occurring within the same country

Supplementary Figure 3: The number of studies sequenced in each African country. Twenty different African countries performed ONT sequencing. Some singular studies performed sequencing in multiple locations, and therefore the additive total differs from the total number of studies.

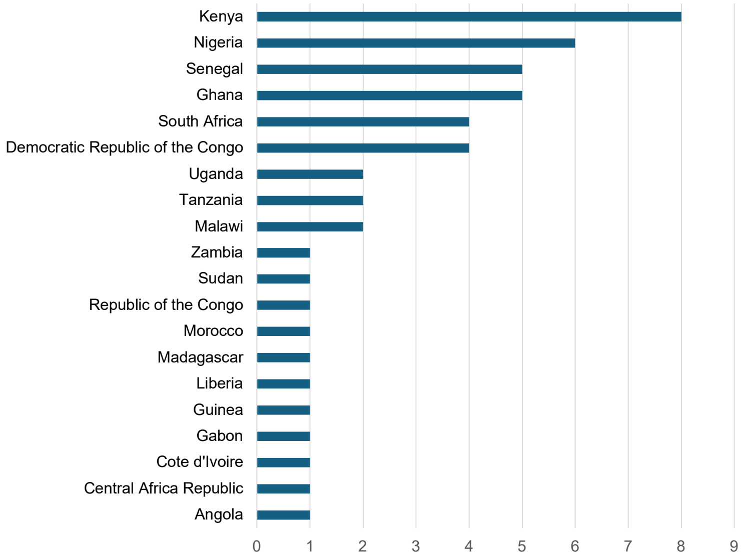

Supplementary Figure 4: Non-SARS-CoV pathogens detected using ONT in Africa in studies that targeted a specific pathogen of interest. Results are grouped by pathogen type (column 1), then sub-categorized by genus (for bacteria, fungi, and parasites) or illness-category (for viruses) in column 2. Individual studies are listed according to author (column 4), year (column 5) and study size (column 6). Results are listed in alphabetical order. Authors designated with an asterisk indicates the study examined more than one pathogen, and therefore the author and associated study is listed more than one time in the table.

Pathogen Type	Genus or Category	Species	Author	Year	Study Size
**Bacteria**	*Acinetobacter*	*A. baumanii*	Moyo	2021	<10
			Odih	2023	11 to 99
			Sanchez-Urtaza	2023	<10
		A. nosocomialis	Snyman	2021	<10
	Bordetella	*B. trematum*	Tchan*	2023	<10
	Clostridioides	*C. difficile*	Darkoh	2022	<10
	Escherechia	*E. coli*	Milenkov	2023 (pre-print)	11 to 99
			Tegha	2021	11 to 99
			Lupande-Mwenebitu*	2021	<10
	Helicobacter	*H. pylori*	Mwangi	2020	<10
	Klebsiella	*K. pneumoniae*	Ahmed	2021	<10
			Ofosu-Appiah	2024	<10
			Pedersen	2020	<10
	Neisseria	*N. gonorrhoeae*	Agbodzi	2024	11 to 99
			Juma	2021	11 to 99
		N. meningitidis	Brynildsrud	2018	<10
			Brynildsrud	2019	<10
			MacAlasdair	2021	Not specified
	Providencia	*P. vermicola*	Lupande-Mwenebitu*	2021	<10
	Pseudomonas	*P. aeruginosa*	Olalekan	2023	<10
			Kiyaga	2022	<10
		P. qingdaonensis	Ogoti	2022	<10
	Salmonella	*S. enterica* serovar Typhimurium	Van Puyvelde	2022	<10
	Staphylococcus	Methicillin-resistant *S. aureus*	Monecke	2023	<1
	Vibrio	*V. cholerae*	Ekeng	2021	11 to 99
**Fungi**	*Cryptococcus*	*C. neoformans, C. gattii*	Kebabonye	2023	11 to 99
**Mycobacteria**	*Mycobacterium*	*M. avium subsp. paratuberculosis*	Elmagzoub	2022	11 to 99
		M. tuberculosis	Hall	2023	≥ 100
			Nilgiriwala	2023	11 to 99
			Tafess	2020	≥ 100
		M. ulcerans	Tchan*	2023	<10
**Parasite**	*Plasmodium*	*P. falciparum*	de Cesare	2024	11 to 99
			Girgis	2023	≥ 100
			Hosch	2024	11 to 99
**Virus**	Arboviruses	Chikungunya virus	Bower	2021	11 to 99
			Dieng	2023	11 to 99
			Vairo, 2020	2020	<10
		Dengue virus	Dieng	2022	≥ 100
			Dieng	2023	11 to 99
			Dieng	2024	<10
			Hafsia	2023	<10
			Hill	2019	<10
			Lazaro	2024	<10
			Neto	2022	11 to 99
		Yellow fever virus	Shaibu	2023	11 to 99
	Viral Hemorrhagic Fever	Ebola virus	Hoenen	2016	11 to 99
			Mbala-Kingebeni	2019	<10
			Quick	2016	≥ 100
			Wawina-Bokalanga	2019	<10
		Lassa virus	Kafetzopoulou	2019	≥ 100
		Sudan virus	Balinandi	2023	≥ 100
	Other	Adenovirus	Lambisia	2023	11 to 99
		Hepatitis B virus	Phinius	2024	≥ 100
			Phinius	2023	11 to 99
			Tshiabuila, preprint	2024 (pre-print)	≥ 100
		Hepatitis C virus	Postigo-Hidalgo	2022	11 to 99
		Monkeypox virus	Vandenbogaert	2022	<10
		Norovirus	Makhaola	2021	11 to 99
		Poliovirus	Shaw	2023	Not Specified

Supplementary Figure 5: Summary of studies using ONT for pathogen detection other than SARS-CoV-2 during infectious diseases outbreaks. Studies are grouped by pathogen in alphabetical order.

Pathogen	Author	Year	Sequencing Device	Sample Size	Sequencing Location	Other device used in addition to ONT
Chikungunya virus	Bower	2021	Minion	11 to 99	Same Country	None
	Dieng	2023	Gridion	11 to 99	Same Country	Illumina
	Vairo	2020	Minion	<10	Not Specified	Sanger
Dengue virus	Dieng	2022	Minion	≥ 100	Same Country	None
	Dieng	2024	Mk1c	<10	Same Country	None
	Hill	2019	Minion	<10	Same Country	None
	Lazaro	2024	Mk1c	<10	Not Specified	None
	Neto	2022	Minion	11 to 99	Same Country	None
Ebola virus	Hoenen	2016	Minion	11 to 99	On site	Sanger
	Mbala-Kingebeni	2019	Minion	<10	Same Country	None
	Quick	2016	Minion	≥ 100	On site	None
	Wawina-Bokalanga	2019	Minion	<10	Same Country	None
Lassa virus	Ashcroft	2022	Minion	11 to 99	Same Country and Non-Africa	None
	Kafetzopoulou	2019	Minion	≥ 100	Same Country	Illumina
P. falciparum	de Cesare	2024	Minion and Flongle	11 to 99	Same Country and Non-Africa	None
Monkeypox virus	Vandenbogaert	2022	Minion	<10	Same Country	Illumina
Neisseria meningitidis	Brynildsrud	2018	Minion	<10	Non-Africa	Illumina
Polio virus	Shaw	2023	Minion, Mk1c, gridion	Not Specified	Same Country	Sanger
Sudan virus	Balinandi	2023	Minion	≥ 100	Same Country	Illumina
